# EZH2 in Myeloid Malignancies

**DOI:** 10.3390/cells9071639

**Published:** 2020-07-08

**Authors:** Jenny Rinke, Andrew Chase, Nicholas C. P. Cross, Andreas Hochhaus, Thomas Ernst

**Affiliations:** 1 Klinik für Innere Medizin II, Universitätsklinikum Jena, 07743 Jena, Germany; Jenny.Rinke@med.uni-jena.de (J.R.); Andreas.Hochhaus@med.uni-jena.de (A.H.); 2 School of Medicine, University of Southampton, Southampton SO17 1BJ, UK; A.J.Chase@soton.ac.uk (A.C.); ncpc@soton.ac.uk (N.C.P.C.); 3 Wessex Regional Genetics Laboratory, Salisbury NHS Foundation Trust, Salisbury SP2 8BJ, UK

**Keywords:** EZH2, PRC2, ASXL1, myeloid malignancies, MDS, MPN, CML, mutations

## Abstract

Our understanding of the significance of epigenetic dysregulation in the pathogenesis of myeloid malignancies has greatly advanced in the past decade. Enhancer of Zeste Homolog 2 (EZH2) is the catalytic core component of the Polycomb Repressive Complex 2 (PRC2), which is responsible for gene silencing through trimethylation of H3K27. EZH2 dysregulation is highly tumorigenic and has been observed in various cancers, with EZH2 acting as an oncogene or a tumor-suppressor depending on cellular context. While loss-of-function mutations of EZH2 frequently affect patients with myelodysplastic/myeloproliferative neoplasms, myelodysplastic syndrome and myelofibrosis, cases of chronic myeloid leukemia (CML) seem to be largely characterized by EZH2 overexpression. A variety of other factors frequently aberrant in myeloid leukemia can affect PRC2 function and disease pathogenesis, including Additional Sex Combs Like 1 (*ASXL1*) and splicing gene mutations. As the genetic background of myeloid malignancies is largely heterogeneous, it is not surprising that EZH2 mutations act in conjunction with other aberrations. Since EZH2 mutations are considered to be early events in disease pathogenesis, they are of therapeutic interest to researchers, though targeting of EZH2 loss-of-function does present unique challenges. Preliminary research indicates that combined tyrosine kinase inhibitor (TKI) and EZH2 inhibitor therapy may provide a strategy to eliminate the residual disease burden in CML to allow patients to remain in treatment-free remission.

## 1. Introduction

Myeloid malignancies are clonal hematopoietic disorders of the myeloid lineage, thought to arise from aberrant pluripotent stem cells, which give rise to diverse cell populations, some of which can be defined by specific genetic abnormalities. The current World Health Organization (WHO) classification of myeloid neoplasms and acute leukemia encompasses acute myeloid leukemia (AML) with various related neoplasms and several entities in the myeloproliferative neoplasm (MPN), myelodysplastic syndrome (MDS) and the overlap of myelodysplastic/myeloproliferative neoplasm (MDS/MPN) categories [1]. Our understanding of the significance of epigenetic dysregulation in the pathogenesis of these disorders has greatly advanced in the past decade. Through the improvements of sequencing technology, genetic, transcriptomic and epigenetic analyses have generated a vast amount of information with regards to the role of post-translational histone modification, DNA methylation, RNA splicing and chromosomal organization in health and disease [2]. Post-translational modifications in the form of histone tail modifications are a key mechanism in the regulation of gene transcription and involve amino acid acetylation, phosphorylation and methylation signatures, which are introduced, read and removed by numerous chromatin modifying enzymes [3]. The Polycomb Repressive Complex (PRC)2 is a well-researched example of such an enzyme and consists of multiple core and regulatory proteins. As a complex these proteins influence chromatin structure and thus DNA transcription to selectively silence target genes through the trimethylation of histone H3 at lysine 27 (H3K27me3). As the catalytic core component, Enhancer of Zeste Homolog 2 (EZH2) has been extensively analyzed, particularly because dysregulation of EZH2 is strongly oncogenic and has been observed in various cancers, making this protein an interesting therapeutic target [4]. This review focuses on recent findings on the role of EZH2 dysregulation in myeloid malignancies.

## 2. EZH2—A Key Epigenetic Regulator

**Figure 1:**
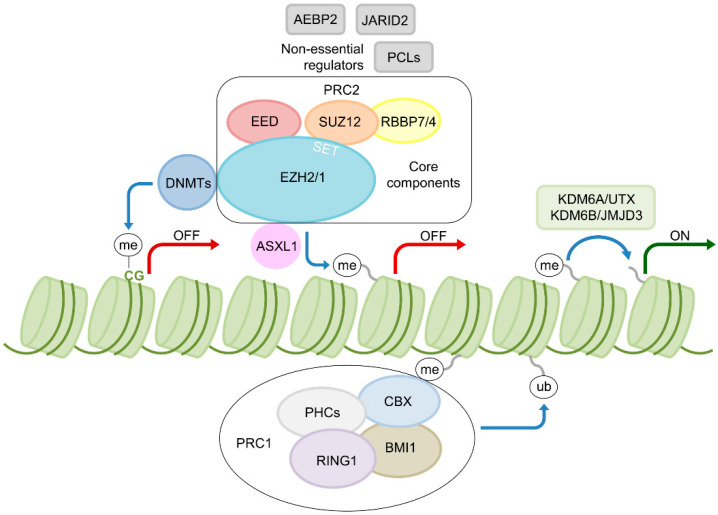
PRC2 and its function in transcriptional regulation. The PRC2 facilitates gene silencing through methylation of H3 lysine 27 and is composed of the core components Enhancer of Zeste Homolog 2 (EZH2), Embryonic Ectoderm Development (EED), Suppressor of Zeste 12 (SUZ12) and Retinoblastoma-binding Protein (RBBP7/4). Other non-essential regulatory proteins, thought to have modulatory and enhancing effects on PRC2 function, include Adipocyte Enhancer-binding Protein 2 (AEBP2), Polycomb-like Proteins (PCLs) and Jumonji and AT-rich Interaction Domain Containing 2 (JARID2) [4,6]. The polycomb complex PRC1, ubiquitinates H2A lysine 119 (H2AK119) and can act downstream of PRC2 to induce gene repression, by binding of trimethylated H3 lysine 27 (H3K27me3) via its Chromobox (CBX) component. Other components include Polycomb Group RING Finger Protein 4 (BMI1), Ring Finger Protein 1 (RING1) and Polyhomeotic Homolog 1 (PHC1) [5]. EZH2 physically interacts with Additional Sex Combs Like 1 (ASXL1) for PRC2 recruitment to target genes [10] and DNA Methyltransferases (DNMTs) to regulate DNA methylation [11]. Demethylation of H3K27 is facilitated through Lysine Demethylase 6A (KDM6A/UTX) and Lysine Demethylase 6B (KDM6B/JMJD3) [12]. me: methylation; ub: ubiquitination; ON/OFF: transcriptional activation/repression of target genes such as Homeobox (*HOX*) genes.

EZH2 belongs to the polycomb group (PcG) proteins. These proteins promote gene silencing through histone modifications. This repressive function of PcG proteins is balanced with the antagonistic activating function of Trithorax-group (TrxG) proteins to regulate gene expression in development and adult tissue homeostasis. In mammals, there are two major complexes of PcG proteins, namely PRC1 and PRC2 [5]. The latter functions as a histone H3 lysine 27 methyltransferase and consists of four core components, namely EZH2, Embryonic Ectoderm Development (EED), Suppressor of Zeste 12 (SUZ12) and Retinoblastoma-binding Protein (RBBP7/4). Other regulatory proteins, which are not essential but are thought to have modulatory and enhancing effects in terms of enzyme function, include Adipocyte Enhancer-binding Protein 2 (AEBP2), Polycomb-like Proteins (PCLs) and Jumonji and AT-rich Interaction Domain Containing 2 (JARID2) [4]. The polycomb complex PRC1 ubiquitinates H2A lysine 119 (H2AK119) and can act downstream of PRC2 to induce gene repression, by binding of trimethylated H3 lysine 27 (H3K27me3) via its Chromobox (CBX) component (Figure 1). Thus, PRC1 and PRC2 share a common set of targets [6]. However, in hematopoietic stem cells (HSC) and progenitor cells (HPC) PRC1 and PRC2 do not necessarily function synergistically in a hierarchical fashion but have opposing roles. This is indicated by the presence of different gene expression signatures and the induction of cell defects or enhanced population activity upon PRC1 or PRC2 abrogation, respectively [7]. In line with these findings, overexpression of EZH2 in HSCs prevents exhaustion of their long-term repopulating capacity in serial transplantation assays, suggesting a role in stem cell senescence [8]. Though EZH2 is an essential subunit of the PRC2, it also has PRC2-independent roles in transcriptional activation and can methylate a number of non-histone protein substrates. However, the contribution of these noncanonical functions and their potential role in cancer pathogenesis remain largely unclear [9].

Additional Sex Combs Like 1 (ASXL1), a protein frequently affected by mutations in myeloid malignancies, has been shown to physically interact with EZH2 and influence PRC2 recruitment in hematopoietic cells, thus regulating gene expression by facilitating PRC2-mediated transcriptional repression of known leukemic oncogenes [10] (Figure 1). Furthermore, demethylation of H3K27 is facilitated through Lysine Demethylase 6A (UTX/KDM6A) [12], an enzyme also recurrently affected by genetic alterations in myeloid malignancies [13]. Other proteins functioning downstream of the PRC2 include DNA Methyltransferases (DNMTs). The catalytic function of DNMTs is to generate 5-methylcytosine (5mC) through the addition of a methyl group to the five-carbon position of cytosine bases in CpG dinucleotides. The 5mC nucleotide serves as a substrate for the successive generation of 5-hydroxymethylcytosine (5hmC), catalyzed by Ten-Eleven Translocation Methylcytosine Dioxygenase (TET) enzymes. These essential epigenetic mechanisms regulate various biological functions, such as cellular differentiation and genome stability [14]. Vireé et al. were able to show that EZH2-mediated H3K27 methylation can influence CpG methylation and involves the direct interaction of EZH2 with DNMTs (Figure 1). This process may contribute to de novo CpG methylation and the maintenance of silenced epigenetic states, e.g., in X-chromosome inactivation [11]. This shows that histone methylation does not function in isolation but is tightly connected to DNA methylation, with PRC2 as a linking element. Moreover, *DNMT3A* is frequently affected by mutations in myeloid malignancies [3]. Overall, PRC2-mediated regulation of gene expression impacts normal hematopoiesis. Various defects along multiple epigenetic regulatory axes can impact PRC2 function and myeloid pathogenesis, with EZH2 playing an important role.

## 3. EZH2 Aberrations in Myeloid Malignancies

In a large number of human cancers, including breast, prostate, and bladder tumors, PRC2 dysregulation is the result of EZH2 overexpression [15,16]. In line with these findings, heterozygous gain-of-function mutations of the amino acid Y641, involving a variety of different substitutions, have been observed in 30% of germinal center-like diffuse large B-cell lymphoma and 10% of follicular B-cell lymphoma cases [17]. These mutations work in conjunction with wild-type EZH2 to increase H3K27me3 levels and are therefore functionally equivalent to EZH2 overexpression [18].

EZH2 is commonly overexpressed in high-risk MDS and AML [19]. However, in 2010, loss-of-function mutations of EZH2 were observed in myeloid malignancies, most frequently affecting patients with MDS/MPN (10–13%), myelofibrosis (13%) and various MDS entities (6%) [20,21]. Taken together, these findings highlight that EZH2 can function as a tumor suppressor and oncogene depending on the cellular context. In contrast to B-cell lymphomas, which are characterized by localized gain-of-function EZH2 mutations, mutations in myeloid malignancies were found to be spread throughout the gene, comprising largely missense, frameshift and nonsense mutations, with missense mutations often located in the CXC/SET or D2 domains. Additionally, mutations were detected in heterozygous as well as homozygous states, with homozygosity often associated with acquired uniparental disomy for chromosome 7q. The survival of patients with homozygous mutations tends to be shorter compared to patients who carry heterozygous mutations [20]. Overall, MPN patients, particularly primary myelofibrosis (PMF) patients, and patients with MDS, MDS/MPN or (secondary) AML who carry EZH2 mutations have a worse prognosis compared to patients with wild-type EZH2 [20,22,23,24]. Nonetheless, EZH2 mutations in MDS are not associated with progression to AML [22,25]. In terms of de novo AML, studies have shown that loss-of-function mutations of EZH2 affect only about 1–2% of patients [26,27]. Although mutations appear to be rare events, there is evidence which indicates that EZH2 mutations might contribute to the pathogenesis of specific cases of childhood AML; for example, Ernst et al. detected a somatic, homozygous in-frame 6 bp insertion within EZH2 exon 20 in a 16-year old patient with a 45,X,t(8;21)(q22;q22) karyotype. However, the incidence of such mutations needs to be assessed in a larger pediatric cohort, particularly in children with t(8;21) [28]. On a cytogenetic level, EZH2 is located at 7q36.1 [29]. In both AML and MDS, this region is frequently affected by loss of chromosome 7 or deletion of 7q—a karyotype associated with an adverse prognosis [30]—perhaps in part as a result of haploinsufficiency or loss of EZH2. Inactivation of other PRC2 components equivalent to EZH2 loss has also been observed in MPN and MDS/MPN patients, e.g., in the form of loss-of-function mutations of *SUZ12* and *EED* [31,32]. Though mutations in these genes occur at a lower frequency compared to EZH2 mutations, they lead to reduced PRC2 histone methyltransferase activity in vitro, underlining that PRC2 function may be compromised through mutations of distinct genes [31]. EZH2 mutations have also been observed in chronic myeloid leukemia (CML) [33]. While other myeloid malignancies show a great degree of genetic heterogeneity within and between the various disease entities, affecting genes involved in transcriptional and cell cycle regulation, the splicing machinery, cell signaling and of course epigenetic regulation [34], CML is principally characterized by a single molecular-cytogenetic aberration—the Philadelphia (Ph) chromosome, which arises from a reciprocal translocation between chromosomes 9 and 22 [35]. This translocation leads to a gene fusion between the Breakpoint Cluster Region (*BCR*) and the Non-Receptor Tyrosine Kinase ABL Proto-Oncogene 1 (*ABL1*), coding for a constitutively active tyrosine kinase, which essentially accounts for CML pathology [36]. Recently, using an NGS panel of 25 leukemia-associated genes, Schmidt et al. detected myeloid leukemia-associated mutations in addition to the characteristic *BCR-ABL1* in 43% of chronic phase patients (6/14) with clonal cytogenetic abnormalities, in Ph-negative cells during tyrosine kinase inhibitor (TKI) treatment. One patient carried an EZH2 mutation (H297Q) at 14% variant allele frequency (VAF). At the point of CML diagnosis the mutation was found at only 1% VAF, indicating that the EZH2-positive clone was likely suppressed by Ph-positive cells and unmasked during TKI therapy [33]. Various other patterns of clonal evolution involving persistence, clearance and acquisition of mutations in addition to *BCR-ABL1*, have been observed in this and other studies. Though epigenetic regulators such as ASXL1 are frequently affected by mutations, all in all, EZH2 mutations seem to be rare events in CML [33,37,38]. However, recently, it was shown that expression levels of EZH2 in bone marrow mononuclear cells (BMMNCs) isolated from CML patients were significantly higher compared to expression in BMMNCs isolated from healthy volunteers as well as individuals with Ph-negative MPNs [39]. Additionally, a study comparing the gene expression profiles of human CML stem cells with normal HSCs found an upregulation of EZH2 in all CML disease phases. Moreover, an enrichment of EZH2 targets was observed, suggesting enhanced activity in leukemic stem cells (LSCs) [40]. Overall, EZH2 aberrations in the form of mutations or altered expression play an important role in the pathogenesis of various myeloid malignancies. Mutant EZH2 seems to be associated with an unfavorable prognosis indicating that mutations act as drivers rather than bystanders. The molecular consequences of the multitude of aberrations that have been observed thus far have been partially investigated but need further attention.

## 4. Mechanisms of EZH2 Dysregulation

In general, nonsense and frameshift mutations are considered to be mostly inactivating. However, pathogenic consequences of missense mutations are frequently unclear, creating diagnostic and prognostic uncertainties, especially in light of the increasing use of NGS in clinical settings. The first study to identify loss-of-function mutations of EZH2 in myeloid malignancies utilized a histone methylase transferase (HMT) assay to confirm EZH2 inactivation conferred by missense mutations [20]. However, the study only focused on the CXC/SET domain variants Y731D, C576W, Y646C and R690C. To understand the role of mutations outside the CXC/SET domain a recent study conducted by Chase et al. focused primarily on variants clustered within the D1 and D2 domains of EZH2 [41]. In this study, two variants detected outside the D1, D2 and CXC/SET domains were found not to inhibit functional activity of the PRC2, suggesting they are bystander mutations or benign constitutional variants. However, all analyzed mutations within the D1 domain (L128F, P132S, M134K, F145C, K156E) resulted in partial or complete loss of methylation activity. A mechanistic explanation for this has been provided by work showing that D1 mutations are likely to disrupt a stimulation-responsive motif, occupying most of region D1, which is critical for allosteric activation of EZH2 by EED [42]. In contrast, mutations within region D2 (E249K, L252V, A255T, R288Q, R298L), which overlaps with exon 8, were not found to directly affect EZH2 protein activity. Instead, testing for splicing abnormalities using qRT-PCR and a mini-gene splicing assay showed exon 8 skipping for these D2 variants with production of an out-of-frame EZH2 mRNA [41]. This is in agreement with a previous study investigating EZH2 splicing abnormalities in MDS, which found a high degree of splicing deregulation between and within patients involving multiple exons [43], indicating that EZH2 splicing abnormalities play an important role alongside loss-of-function mutations in PRC2 dysregulation.

In this context, it is important to highlight that mutations of genes that encode the spliceosome are very common in myeloid malignancies. They occur frequently in MPN-derived secondary AML [44], in MDS and the MDS/MPN overlap category, with over half of chronic myelomonocytic leukemia (CMML) patients affected. Mutations predominantly affect genes that play a role in the 3′-splice site recognition during pre-mRNA processing, including Splicing Factor 3b Subunit 1 (*SF3B1*), U2 Small Nuclear RNA Auxiliary Factor 1 (*U2AF1*), Zinc Finger CCCH-Type, RNA-binding Motif and Serine/Arginine Rich 2 (*ZRSR2*) and Serine and Arginine Rich Splicing Factor 2 (*SRSF2*) [45,46,47,48]. It has been shown that *SRSF2* and *U2AF1* mutations are associated with an adverse outcome [44,49], cause aberrant function of the spliceosome machinery and lead to dysfunctional hematopoiesis, as they can change the splicing patterns of proteins, altering their function, rather than causing splicing failure altogether [50,51]. *SRSF2* mutations in particular affect EZH2 expression, as they do not confer a loss-of-function but change sequence-specific RNA-binding activity altering the recognition of splicing enhancer motifs. This leads to aberrant splicing and preferential expression of a specific EZH2 isoform, which is subjected to nonsense mediated decay. As a result, cells bearing mutant *SRSF2* have diminished expression of EZH2 and a lower abundance of H3K27me3 [50]. This shows that the prevalence of PRC2 dysfunction is greater than the frequency of EZH2 mutations and that even mutations of genes that do not encode components of the PRC2 can affect its function and thus influence H3K27 methylation levels [52]. The polycomb-related gene *ASXL1*, for example, is frequently mutated in MDS, MPN and AML, and mutations are associated with an adverse outcome [22,53,54]. Loss of ASXL1 reduces global H3K27me3 levels and activates the expression of posterior *HOXA* genes, such as *HOXA9*, known to play a role in leukemogenesis. This is the result of impaired recruitment of PRC2, which leads to myeloid transformation despite the reduced repopulating capacity of ASXL1-deficient HSCs [10,55].

In CML, EZH2 dysregulation in the form of overexpression is associated with BCR-ABL1 activity. This was shown in vitro through *BCR-ABL1* transduction of Ba/F3 cells, which resulted in a simultaneous increase of EZH2 levels and Signal Transducer and Activator of Transcription (STAT) 5 phosphorylation, suggesting that BCR-ABL1 positively regulates the expression of EZH2 via STAT5 signaling. To support these findings, chromatin immunoprecipitation assays have revealed that STAT5A locates in the promoter region of EZH2, leading to increased transcription in leukemic cells [39]. Moreover, TKI treatment of LSCs in vitro results in decreased EZH2 levels, while EZH1 levels are maintained. These results also point towards a kinase dependence of EZH2 expression [56].

While the mechanisms of EZH2 and PRC2 dysregulation in leukemia development and progression are progressively better understood, it is important to view aberrations in the bigger picture of a heterogeneous molecular background with multiple genetic lesions contributing to pathogenesis in the majority of myeloid malignancies. Researchers have therefore focused increasingly on entire mutation landscapes to investigate how various aberrations work together in different disease entities, ultimately to aid prognostic and therapeutic advancements.

## 5. Mutual Exclusion and Concomitance of EZH2 and Other Leukemia-Associated Mutations

EZH2 loss-of-function mutations tend to be mutually exclusive with *SRSF2* and *U2AF1* mutations in patient samples [47,52]. Mutual exclusion of such driver mutations can occur if two hits in the same pathway are either not selected because they do not provide a growth advantage (epistasis) or because they compromise the life of the cell (synthetic lethality) [57]. Mutual exclusion of splicing and EZH2 mutations is therefore a plausible observation as mutations of genes encoding the splicing apparatus can affect the same regulatory axis as EZH2 mutations, as detailed above. Similarly, *UTX* and EZH2 mutations have not been detected simultaneously [52,58]. Since UTX functions as a H3K27-demethylase loss of UTX activity might be equivalent to EZH2 gain-of-function or overexpression. In contrast to EZH2 mutations, *UTX* mutations seem to be acquired later in malignant evolution as they are detected in more aggressive forms of CMML and secondary AML derived from CMML [58]. Considering that EZH2 overexpression has been observed at progressively increasing levels from low-risk, to high-risk MDS, to AML [19] it makes sense that loss-of-function *UTX* mutations (equivalent to EZH2 overexpression) are late events in leukemogenesis and that the mutual exclusion of loss-of-function EZH2 and *UTX* mutations is likely the result of repression of leukemic growth through opposing effects. Overall, mutations of factors with opposing enzymatic activity can contribute to leukemogenesis, highlighting once again the dual role of the PRC2 in cancer, as both an oncogenic and tumor suppressive. In either situation, greater steady state levels of H3K27me3 are induced in malignant cells [58].

In terms of co-operativity, patients frequently present with co-occurring heterozygous mutations of EZH2 and *ASXL1* [10,22,52,59]. Given that PRC2 dysregulation can result from mutant *ASXL1*, haploinsufficiency for multiple genes that regulate PRC2 function can cooperate in leukemogenesis through additive alterations [10]. In addition to frequent *ASXL1* mutations, a significant association of EZH2 mutations with RUNX Family Transcription Factor 1 (*RUNX1*) mutations has been observed in MDS and MDS/MPN patients [25,48,60]. In a mouse model established by Sashida et al., it was shown that EZH2 loss in HSCs significantly promotes *RUNX1*S291fs-induced MDS. Though the proliferative capacity of *RUNX1*S291fs/EZH2-null MDS cells was hampered, the study revealed that MDS bone marrow impairs normal hematopoietic cells via the activation of inflammatory cytokine responses, including the interleukin-6 (IL-6) pathway, which have been shown to impair normal HSCs in vivo, thereby promoting the survival and proliferation of MDS clones [61]. Another study conducted by Booth et al. focused on inactivation of EZH2 and RUNX1 in progenitor rather than stem cells and found that combined inactivation enhances proliferation of early thymic progenitors in mice. Additional RAS-signaling pathway activation in form of an internal tandem duplication of Fms Related Receptor Tyrosine Kinase 3 (*FLT3*-ITD), frequently detected in AML and associated with a worse prognosis, resulted in aggressive lympho-myeloid leukemia [62]. EZH2 mutations also frequently co-occur with TET2 mutations [47,48,60]. Murine analyses conducted by Muto et al. revealed that EZH2 or TET2 deficiency alone is sufficient to induce an MDS/MPN phenotype with lethality during long observation periods, while depletion of both EZH2 and TET2 synergistically advances myelodysplasia and accelerates the progression of both MDS and MDS/MPN. Genome analyses in HPCs revealed partial compensation of EZH2 deletion through EZH1, as developmental regulator genes were kept transcriptionally repressed, while a number of direct and indirect polycomb targets became transcriptionally active [63]. Subsequent functional analyses conducted by Hasegawa et al. revealed changes in the epigenome resulting from TET2 insufficiency followed by loss of EZH2. Interestingly, DNA hypermethylation patterns observed in HSCs/HPCs of mice with aberrant TET2 and EZH2 were largely distinct from those observed in single mutant mice. While TET2 insufficiency alone caused enhancer hypermethylation, methylation patterns changed in combination with the loss of EZH2 and correlated with transcriptional repression in MDS cells, but not in cells carrying aberrant TET2 only [64]. These results show the effects of combined TET2 and EZH2 dysfunction on the cooperative remodeling of the epigenome in MDS pathology and suggest that the order of mutation acquisition plays an important role in the progression of the disease [63,64]. Mouse models of MPN have also shown that EZH2 plays a tumor suppressive role in PMF, as additional deletion of EZH2 in mice carrying the activating *JAK2* V617F mutation promotes the development and progression of myelofibrosis, resulting in reduced survival, increased platelet and neutrophil counts and more advanced disease [65]. The loss of EZH2 in *JAK2* V617F hematopoietic cells was shown to reduce H3K27me3 levels with an increase in H3K27 acetylation and the resulting activation of PRC2 target genes including High Mobility Group AT-Hook 2 (*HMGA2*), an oncogene previously implicated in PMF and other myeloid neoplasms [66,67]. In AML mouse models induced by the Mixed Lineage Leukemia (*MLL*)-*AF9* translocation, EZH2 inactivation resulted in significantly reduced leukemia-initiating cells and enhanced differentiation [68]. Deletion of *EED* results in complete loss of PRC2 function and complete inhibition of leukemia growth. These findings indicate that the presence of EZH2 is not strictly required for *MLL-AF9* induced AML probably due to EZH1 compensation [69]. However, EZH2 does seem to have an oncogenic role in AML and contributes to the pathogenesis through silencing of PRC2 target genes to impede differentiation of leukemic stem cells [68,69].

The majority of patients with myeloid malignancies carry multiple somatic mutations that drive clonal evolution. As shown above, mouse models have often focused on paired genetic mutations and the generation of these models used to be both time consuming and costly. With the advancement of CRISPR-Cas9 genome editing, the generation of murine models has become more efficient and increasingly complex to appropriately reflect and model the genetic heterogeneity seen in human leukemia [70,71]. Particularly promising are recent approaches, which focus on method optimization of CRISPR/Cas9 genome engineering on primary human HSCs and HPCs, followed by transplantation into immune deficient mice to generate models of clonal hematopoiesis and neoplasia [72,73]. Such approaches will no doubt help researchers to better understand how mutations co-operate in leukemic disease, with the ultimate goal of identifying druggable targets and advancing personalized treatment strategies, based on each patient’s individual molecular background.

## 6. Kinetics of EZH2 Mutations

**Figure 2:**
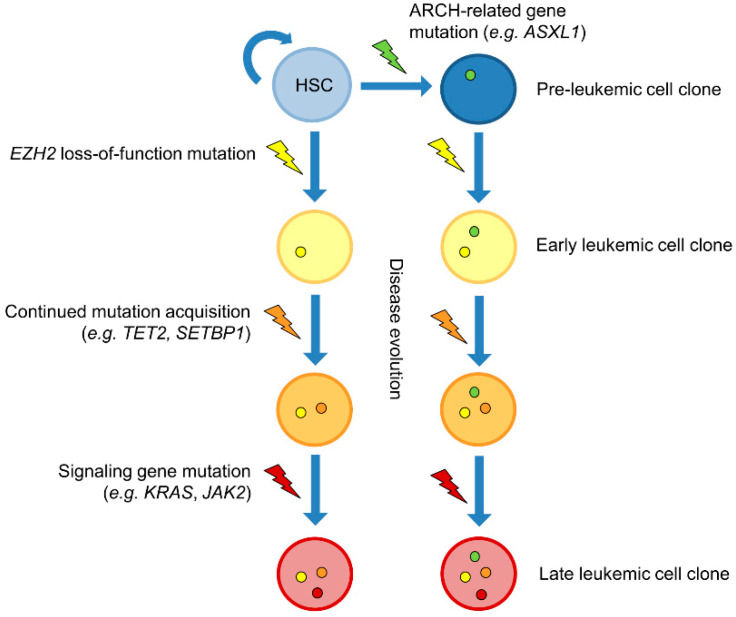
Clonal evolution of myeloid malignancies with loss-of-function mutations of EZH2. Loss-of-function mutations of Enhancer of Zeste Homolog 2 (EZH2) are thought to be early events in clonal evolution of myeloid malignancies. Studies have shown that mutations of EZH2 can precede the acquisition of mutations of other epigenetic regulators such as Ten-Eleven Translocation Methylcytosine Dioxygenase 2 (TET2). Signaling gene mutations usually occur later in disease evolution. Additional Sex Combs Like 1 (*ASXL1*) mutations have been shown to precede the acquisition of EZH2 mutations [20,60,74]. Unlike EZH2 mutations, *ASXL1* mutations have been shown to be involved in age-related clonal hematopoiesis (ARCH) [75,76,77], suggesting loss-of-function EZH2 mutations are highly oncogenic and frequently involved in leukemia initiation. HSC: Hematopoietic stem cell; *JAK2*: Janus Kinase 2; *KRAS*: Kirsten Rat Sarcoma Viral Oncogene Homolog; *SETBP1*: SET-binding Protein 1.

As the genetic background of cancer phenotypes has been unraveled over the past years, researchers have become more aware of the importance of the order of mutation acquisition, i.e., clonal evolution—a natural process in cancer pathology, influenced by therapeutic intervention. When EZH2 mutations were first detected in myeloid malignancies, it was assumed that they are early events in the disease process, as they were detected in patients with refractory anemia, a relatively early stage of MDS [20]. A study conducted by Jankowska et al. suggested EZH2 mutations were early events in malignant evolution as they were detected in patients with CMML-1, the prognostically favorable CMML subgroup [58]. Colony progenitor assays have confirmed the early acquisition of mutant EZH2 in a patient with CMML who carried two TET2 mutations in addition to an EZH2 mutation [20] and in three patients with atypical CML who carried concomitant SET-binding Protein 1 (*SETBP1*) mutations, among other myeloid leukemia-associated mutations. One patient with MDS/MPN-unclassifiable carried an *ASXL1* mutation, which was found to precede the acquisition of two EZH2 mutations [60]. This is in line with a comprehensive study conducted by Mossner et al., where the clonal evolution/order of mutation acquisition was investigated in MDS and CMML patients. Overall, the study identified that founding mutations frequently affect genes involved in the regulation of DNA methylation (TET2, *DNMT3A*), RNA splicing (*SF3B1*, *ZRSR2*) or chromatin remodeling (*ASXL1*, EZH2). In a CMML-1 patient, primary acquisition of an EZH2 mutation was detected, followed by a TET2 mutation and finally signaling pathway mutations of Kirsten Rat Sarcoma Viral Oncogene Homolog (*KRAS*) and Janus Kinase 2 (*JAK2*) [74]. This confirms previous findings that suggest that signaling genes are often affected later on in disease progression [45,60]. In three MDS cases with concomitant *ASXL1* and EZH2 mutations (among other mutations), investigated by Mossner et al., the acquisition of mutant *ASXL1* mutations preceded the EZH2 mutation [74]. *ASXL1* mutations have previously been implicated in age-related clonal hematopoiesis (ARCH). Exome-analyses have identified ARCH in healthy individuals through the detection of mutations linked to myeloid neoplasms. Mutations were associated with an increased risk of hematologic cancer and cardiovascular disease. Healthy younger individuals (<50 years of age) were rarely affected (<1%), whereas about 10% of individuals over 65 years of age showed ARCH. Similar genes have been identified, with the majority of mutations affecting epigenetic modifiers. However, EZH2 mutations do not seem to be involved in ARCH [75,76,77], implying that they are strongly associated with an overt neoplastic state. Thus, EZH2 mutations could be the initiating factor for manifestation of myeloid malignancy or provide a growth advantage necessary for further clonal expansion to outcompete normal HSC clones, in cases where ARCH genes such as *ASXL1* are involved [60] (Figure 2).

Generally, mutational landscapes of myeloid malignancies are defined by complex patterns of molecular heterogeneity, not only between and within the various disease entities, but also on a cellular level, as most patients carry diverse leukemic clones [60,74]. In line with this heterogeneity, the clonal architecture shows frequent branching events, while therapeutic intervention reshapes mutational patterns, altering clonal evolution. In the majority of MDS cases, earlier clones survive therapy [74], highlighting the importance of therapeutic targeting of primary lesions.

## 7. EZH2—A Promising Therapeutic Target

As EZH2 enzymatic gain-of-function is associated with various cancer types, several selective EZH2 inhibitors have been developed and investigated, with Tazemetostat as the first inhibitor approved in early 2020 by the FDA to target epithelioid sarcoma [9,78]. EZH2 loss-of-function, however, provides a more challenging therapeutic hurdle. A recent study conducted by Goellner et al. showed that loss of EZH2 and the resulting reduction of H3K27m3 levels causes acquired drug resistance towards TKI and cytotoxic drugs in AML [79]. Low protein levels of EZH2 correlated with a poor prognosis in patients. The researchers were able to show that suppression of EZH2 protein expression induced chemoresistance in AML cell lines, primary cells and humanized mouse models. Furthermore, low EZH2 levels resulted in a depression of *HOX* genes and subsequent *HOXB7* and *HOXA9* knockdown in resistant cells improved sensitivity to TKIs and cytotoxic drugs. Loss of EZH2 expression in resistant cells and primary blasts from relapsed AML patients was associated with CDK1-dependent phosphorylation of EZH2 at Y487. In drug resistant cells, this interaction was stabilized by Heat Shock Protein 90 (HSP90) leading to proteasomal degradation of EZH2. HSP90, CDK1 and proteasome inhibitors prevented this degradation, decreased *HOX* gene expression and restored drug sensitivity. Finally, patients with reduced EZH2 levels responded to the addition of the proteasome inhibitor bortezomib to the standard therapy with DNA synthesis inhibitor cytarabine with improved EZH2 expression and blast clearance. These results suggest restoration of EZH2 protein as a viable approach to overcome treatment resistance in some AML patients [79] and illustrate that approaches aimed at restoring H3K27me3 levels and consequently chromatin repression may yield therapeutic benefit in other myeloid malignancies as well. One such approach could be aimed at the inhibition of H3K27 demethylases [52]. It has been shown that demethylases such as KDM6A (UTX) and KDM6B (JMJD3) are targetable with small molecules to modulate histone methylation signatures [80]. More recently, a study conducted by Li et al. was able to show that increased *KDM6A* expression correlates with a poor survival in AML patients. Using GSK-J4, a KDM6B-specific inhibitor to treat the primary cells from AML patients and cell lines, resulted in increased H3K27me3 levels and reduced proliferation and colony-forming ability, and attenuated disease progression in a humanized AML mouse model. GSK-J4 application resulted in downregulation of DNA replication and cell cycle pathways, as well as *HOX* genes, which were enriched for H3K27me3 in transcription start sites as revealed by ChIP-qPCR [81]. Interestingly, Ezponda et al. not only showed that loss of UTX/KDM6A leads to changes in the transcriptional profile of multiple myeloma (MM) cells contributing to the pathogenesis of the disease, they also revealed an increased in vitro and in vivo sensitivity of *UTX*/*KDM6A*-mutant cells to EZH2 inhibition, which rebalanced H3K27me3 levels of specific genes, revealing therapeutic strategy in MM cases harboring *UTX* loss-of-function mutations [82]. It is therefore conceivable that the opposite application of an UTX/KDM6A inhibitor in cases with loss-of-function EZH2 might be a viable therapeutic strategy after appropriate investigation.

Alternatively, targeting genes which are derepressed as a result of EZH2 or PRC2 loss-of-function, such as *HOXA9* may provide a successful therapeutic strategy. Inhibition of HOXA9 is achievable either on an epigenetic level through inhibition of expression or directly through inhibition of the HOXA9 transcription factor function, i.e., through targeting protein/protein or protein/DNA interactions. A number of HOXA9 inhibitors such as HXR9 are already under clinical evaluation for patients with AML, where the role of HOXA9 in leukemogenesis was first described [83]. There may also be therapeutic potential for exploring synthetic lethality, where there is mutational loss of EZH2. In mice, EZH1 has been shown to compensate for loss of EZH2 by repositioning of EZH1 to EZH2 targets [63,84]. In two mouse models, this compensatory activity of EZH1 has been found to be essential for the maintenance of hematopoiesis [84,85]. In addition to demonstrating a potential EZH1/EZH2 synthetic lethality, Gu et al. have identified a potential metabolic susceptibility. BCAT1, a transaminase for branched-chain amino acids, is upregulated by EZH2 loss in mouse models and human myeloid neoplasm samples. In a mouse model, BCAT1 inhibition was shown to preferentially target EZH2-deficient cells compared to EZH2 wild-type cells, indicating that EZH2 loss may induce an exploitable dependency on BCAT1 [85].

Another interesting aspect of EZH2 as a therapeutic target is the potential application of specific inhibitors in well responding CML patients, where the primary goal is not to treat overt disease but to eliminate residual LSCs. Previous studies have concluded that CML LSCs are not “oncogene-addicted” and serve as a reservoir to drive relapse or resistance; thus, merely targeting BCR-ABL1 kinase activity using TKI would not eliminate them, which necessitates combined treatment strategies [86,87]. Subsequently, it has been shown that EZH2 overexpression seems to be characteristic for CML LSCs as detailed above. Analyses focused on CML cell lines, primary cells, as well as mouse models, suggest that combined TKI treatment and EZH2 inhibition can delay leukemia development, extend survival and decrease the LSC burden through upregulation of H3K27me3 targets. Furthermore, normal HSCs and progenitor cells were largely resistant to treatment, probably due to EZH1 compensation [40,56]. Thus, TKI treatment combined with EZH2 inhibition could potentially overcome the residual disease burden to allow patients to maintain long-term treatment-free remission.

Overall, EZH2 aberrations and PRC2 dysregulation seem to be extremely prevalent in myeloid leukemia. With a dual tumor suppressive and oncogenic role of EZH2, caution is warranted when modulating PRC2 function, and thus, H3K27 methylation in therapeutic strategies. Nonetheless, as a key epigenetic regulator appearing largely early in disease evolution, targeting EZH2 dysfunction directly or indirectly seems to be a promising approach for a large number of patients.
